# Special Issue “Diagnosis and Management of Skin Diseases, Related Disorders and Their Comorbidities”

**DOI:** 10.3390/diagnostics15070897

**Published:** 2025-04-01

**Authors:** Alin Laurentiu Tatu, Lawrence Chukwudi Nwabudike

**Affiliations:** 1 Clinical Department, Faculty of Medicine and Pharmacy, Dunarea de Jos University, 800008 Galati, Romania; 2 Dermatology Department, Saint Parascheva Clinical Hospital of Infectious Diseases, 800179 Galati, Romania; 3 Multidisciplinary Integrated Center of Dermatological Interface Research (MICDIR), 800010 Galati, Romania; 4 N. Paulescu National Institute of Diabetes, 030167 Bucharest, Romania; chukwudi.nwabudike@live.com

Dermatology is a continuously evolving specialty and touches on every part of the field of medicine. Our Special Issue is aimed at the examination of this continuous evolution and the vastness of our discipline. This Issue will be a collection and presentation of data on the latest developments in the field of dermatology and is intended to be a continuation of articles previously published by the Guest Editors [1,2].

Patients with lichen planus may suffer from mucosal disease, including oral lichen planus (OLP). In district hospitals, lichen planus in all its forms affects about 4% of patients. A single-centered Spanish study with a cohort of 275 OLP patients and 275 controls set out to determine if there was any link between OLP and prediabetes. The diagnosis of prediabetes was based on the American Diabetes Association’s criterion of blood sugar levels of 100–125 mg/dL. The authors found that OLP was significantly associated with prediabetes and with oral antidiabetic medication (i.e., overt type 2 diabetes, as inferred from oral medication use). The study authors concluded that it would be reasonable to check glycemia levels in patients with OLP.

Non-melanoma skin cancers continue to be challenging to diagnose and treat, partly due to their heterogeneity as a group and partly due to their protean manifestations, which sometimes mimic those of other cutaneous disorders. A Romanian study set out to address these difficulties with the use of ultrasound. Their cohort comprised 31 patients with head and neck tumors, who were assessed using 13, 20, and 40 MHz ultrasound transducers, as well as histopathology. The authors found that the 13 MHz transducers offered larger, but less detailed images and may be more useful for large tumors. More detailed images were produced by the 20 and 40 MHz transducers, which were able to discern hyperechoic spots; this could be useful in the diagnosis of basal cell carcinomas, according to the study authors.

Pseudoxanthoma elasticum (PXE) is a rare autosomal recessive disorder, which often begins in the skin. Genetic testing may aid in diagnosis. A Hungarian cohort (*n* = 5) was assessed using autofluorescence (AF) to determine potential diagnostic features of PXE. The authors found that high-intensity AF signaling using a 450 nm LED light device was suitable for patients with PXE and concluded that this process might represent a useful non-invasive method for the early diagnosis of PXE.

Artificial intelligence is increasingly being used in diagnostics. Some authors trained a self-developed neural network to diagnose actinic keratosis. They found that their artificial intelligence program performed better than traditional convoluted neural networks, requiring only 123 s of training.

Psoriatic arthritis can be a challenging disorder to diagnose and to treat. Magnetic resonance imaging (MRI) is increasingly used in the diagnosis of this disorder, as opposed to traditional radiographs. MRI findings of psoriatic arthropathy were reviewed. Bone erosion, the inflammation of vertebral bodies, and spondylodiscitis were amongst the vertebral issues identified in the literature. In addition, ankylosis, joint erosion, enthesitis, capsular inflammation, and fat metaplasia were notable MRI findings at the sacroiliac joint level. These findings may aid dermatologists in the diagnosis of psoriatic arthritis without using overt skin lesions when there is a suspicion of disease.

A group of authors carried out an update on the clinical presentation, differential diagnosis, and diagnostic characteristics of atypical dermatofibroma. They were able to provide data to enable the differentiation of this disorder from other malignant disorders, especially amelanotic melanoma.

Correlations between atopic dermatitis and other skin disorders were identified in a review by another group of authors [3]. They also reviewed the clinical aspects of atopic dermatitis itself, including the ocular manifestations of the disorder, such as blepharitis, keratoconus, and retinal detachment. Correlations were also found between atopic dermatitis and cutaneous infections, as well as non-melanoma skin cancer and respiratory disorders.

In another paper, a case of metastatic melanoma treated with various therapy modes, including immune-checkpoint inhibitors, was presented. The authors reviewed the potential benefits to patients and the adverse effects of multimodal therapy on this complicated disorder, as well as potential approaches to these side effects [4].

A rare association of cutaneous melanoma and glioblastoma was presented as a case report, with a review of the literature. Potential genetic and familial causes were explored. Melanoma may have protean manifestations and multiple associations, as this case report and review shows. Its regressing phase may also resemble collision tumors, thereby intensifying the challenge.

A rare case of peripheral T-cell lymphoma not otherwise specified (PTCL-NOS) was reported. The diagnosis, therapy, and—happily—its subsequent remission were documented. The authors noted the potential for this form of lymphoma to be misdiagnosed as mycosis fungoides or Sézary syndrome. The importance of arriving at the right diagnosis cannot be understated, as the authors remind us, since PTCL-NOS can become fatal very rapidly, while mycosis fungoides is generally an indolent disorder.

The combination of a rare tumor with a more commonly encountered disorder was reported in another paper, namely a case of somatostatinoma and neurofibromatosis type 1. The patient in this case was a 50-year-old, the usual age for the manifestation of somatostatinoma. Metastases had occurred at the time of diagnosis. Neurofibromatosis has protean manifestations, including orthopedic ones. Thus, such combinations may prove challenging to diagnose.

This Special Edition offers 11 diverse and stimulating manuscripts. The significance of general internal medicine in dermatology is illustrated in a significant proportion of these articles. Lichen planus continues to be important in this regard and there are continuous updates to its diagnosis and increasing therapeutic possibilities at our disposal. These broadening therapeutic possibilities may also be applied in the therapy of psoriasis.

## Data Availability

Not applicable.
